# Evaluation of mandibular motion in adolescents with skeletal class II division 1 malocclusion during mandibular advancement using clear functional aligners: a prospective study

**DOI:** 10.1186/s12903-024-04082-3

**Published:** 2024-03-09

**Authors:** Qiuyue Wu, Yueying Zhang, Hua Xiao, Jiajing Zheng, Tianlu Jiang, Yusen Du, Meng Cao, Feifei Li

**Affiliations:** State Key Laboratory of Oral & Maxillofacial Reconstruction and Regeneration, Shaanxi Clinical Research Center for Oral Diseases, Department of Orthodontics, National Clinical Research Center for Oral Diseases, The Third Affiliated Hospital of Air Force Medical University, Xi’an, Shaanxi 710032 People’s Republic of China

**Keywords:** Neuromuscular function, Orthodontic treatment, Mandibular motion, Skeletal class II malocclusion, Functional orthodontic appliance

## Abstract

**Background:**

This study aimed to evaluate the characteristics of mandibular protrusive condylar trajectory in adolescents with skeletal Class II Division 1 malocclusion and the changes of condylar trajectory during mandibular advancement (MA) treatment using clear functional aligners.

**Methods:**

This prospective study consisted of a cross-sectional study and a longitudinal study. In cross-sectional study, sixty-one adolescents were divided into two groups: Class I (*n* = 30) and Class II Division 1 (*n* = 31). The condylar trajectory was measured and compared using the Mann–Whitney U test. The longitudinal study was the MA treatment group using clear functional aligner and consisted of 16 participants from Class II Division 1group. The condylar trajectory was collected at three-time points: pre-treatment (T1), during MA treatment at approximately 3 months (T2, 105.6 days average), and at the end of MA treatment (T3, 237.6 days average). The changes at T1, T2, and T3, as well as the symmetry between the left and right condyles across all groups, were examined using the Wilcoxon paired test.

**Results:**

A greater increase in the anteroposterior displacement and space displacement during protrusive movements was observed in the Class II Division 1 group compared with that in the Class I group, with a large difference being observed in the left and right condylar movements. The condylar anteroposterior displacement and space displacement decreased significantly at T2 and increased significantly at T3; however, no significant difference was observed between T1 and T3. A significant difference was observed between the condylar movement on the left and right sides at T1; however, no significant difference was observed at T2 and T3.

**Conclusions:**

Adolescents with Class II Division 1 malocclusion had higher protrusive capacity than those with Class I. Moreover, their left and right condylar motion was more asymmetric. The range of condyle motion decreased first and then increased during MA therapy, and the left and right condyle movement became more symmetrical, which may be the adaptive response of neuromuscular function to the changes in jaw position.

## Background

Class II malocclusion is frequently encountered orthodontic problem characterized by the presence of a prognathic maxilla, retrognathic mandible, or a combination of the two. Mandibular retrognathism, a major contributing factor, is the most prevalent form of Class II malocclusion. A functional orthodontic appliance designed to advance the mandible during the peak growth and development phase is indicated for these patients, as this phase is believed to coincide with the most significant growth modifications in the mandible and temporomandibular joint (TMJ)[1, 2]. The Herbst, a fixed-functional appliance (FFA), and the Twin Block, a removable-function appliance (RFA), are the most commonly employed functional appliances in clinical practice.

Clear aligners have gained prominence in recent years owing to the advancements in the domains of polymer materials and computer technology. This appliance, which was introduced recently to clinical practice, has garnered significant attention owing to it being accurate, aesthetically pleasing, and easy to maintain[3]. Therefore, clear functional aligners (CFAs) were used for mandibular advancement (MA) to address skeletal Class II Division 1 malocclusion in this study. CFA mimics the action of the twin block owing to the presence of occlusal blocks (typically present between the first molar and premolars of the maxilla and mandible), which can only interlock when the patient moves the mandible anteriorly, thereby correcting crowding and malocclusion simultaneously.

MA treatment achieves the desired outcomes via occlusal reconstruction. Force is generated on soft tissues, such as the masseter muscle, lip, tongue, and cheek, which elicits a neuromuscular response and changes in neuromuscular function[4]. The subsequent changes in the size, direction, and time of force exerted by the orofacial muscles on the dentition and jaw make the neuromuscular environment of the oral and maxillary system more favorable to the normal development and growth of teeth and the maxillocraniofacial structures[4].

The effect of functional appliances depends on the response of the neuromuscular system; thus, it is important to observe the changes in neuromuscular function during MA treatment. Mandibular movement is a complex three-dimensional movement involving the muscles, TMJ, maxilla, mandible, and occlusion that is governed by the nervous system[5]. Thus, mandibular movement can be used to evaluate jaw movement and neuromuscular coordination ability. The study of condylar trajectory is an important index used to evaluate mandibular movement[6].

Previous studies have shown that individuals with Class II malocclusion have increased protrusive capacity than individuals with Class I malocclusion[7]. Longer retrusion was observed in patients with Class II malocclusion who underwent MA treatment, which is consistent with that observed in individuals with untreated Class II malocclusion, rather than that observed in individuals with Class I[8]. This may be related to unconscious habits in such patients[7] and is a manifestation of neuromuscular adaptation[8]. Thieme et al[6]. reported that neuromuscular changes in mandibular movements during treatment depend on different parameters and that one-third of the patients undergoing functional orthodontic therapy exhibited no significant changes in neuromuscular control post-treatment. Thus, monitoring jaw movement during MA, especially the mandibular protrusive movement of the mandible, is an effective method to evaluate the changes in neuromuscular function and assess the effects of therapy.

Therefore, in this study, a mandibular movement analyzer based on the ultrasonic principle was used to analyze the characteristics of protrusive mandibular movement in adolescents with Class II Division 1 and the changes in condylar trajectories during MA treatment with CFA, thereby reflecting the changes in maxillofacial neuromuscular function.

## Methods

This prospective study enrolled patients from the Department of Orthodontics of The Third Affiliated Hospital of Air Force Medical University. Sixty-one participants were enrolled in the trial between 2022 and 2023. Informed consent was obtained from the legal guardians, and ethical approval was obtained from the local research ethics board prior to the commencement of the trial.

### Participants

This study comprised three groups: a control group of Class I, the Class II Division 1 group, and the MA treatment group (MA using CFA). In order to reduce the baseline bias caused by individual differences, all patients in Class II Division 1 group met the requirements for MA therapy.

The control group comprised 30 adolescents with Class I occlusion. The inclusion criteria were as follows: [1] The participants had sound, complete, late mixed or early permanent dentition with bilateral canine and molar Angle Class I relationships; [2] anterior teeth with an overjet of 0–3 mm and an overbite not exceeding one-half of the lower incisors; [3] maximum lateral deviation of 2 mm between the maxillary and mandibular midlines; and [4] adolescent aged 11–16 years in pubertal growth peak, as determined by the cervical vertebrae (stages CS3-CS4)[9].

The Class II Division 1 group comprised 31 adolescents. The inclusion criteria were as follows: [1] Participants with Class II Division 1 malocclusion with at least cusp-to-cusp relationship between molars and canines; [2] skeletal class II relationship (ANB > 5°), retrognathic mandible (SNB <78°), normal or slightly protruding maxilla (78°<SNA<86°); [3] horizontal facial growth pattern (FMA<32°); [4] moderate or severe overjet (overjet>5 mm); [5] late mixed dentition or early permanent dentition; [6] mandibular arch with slight or no crowding; [7] adolescents aged 11–16 years in pubertal growth peak, as determined by the cervical vertebrae (stages CS3-CS4)[9].

The MA treatment group comprised 16 participants selected from the Class II Division 1 group who voluntarily underwent MA treatment using CFA. All patients underwent examinations of the mandibular movement at different stages.

The exclusion criteria were as follows: [1] significant tooth anomalies, such as retained crowns or roots, missing teeth, or congenitally missing teeth; [2] history of undergoing maxillofacial surgery or orthodontic treatment; [3] history of TMJ disorders, discomfort, or muscle-related issues in the masticatory muscles or around the joint; [4] history of cleft lip or palate during childhood; [5] history of undergoing surgical procedures for the removal of cysts or tumors; and [6] history of systemic disease.

### Experimental design

The current study incorporated a cross-sectional study and a longitudinal study. The cross-sectional study consisted of Class I and Class II Division 1 groups. The participants in the Class I and Class II Division 1 groups only underwent mandibular motion measurements pre-treatment. The longitudinal study consisted of an MA treatment group. The participants in the MA treatment group underwent mandibular motion measurements at three time points: pre-treatment (T1), during MA treatment at approximately 3 months (T2, 105.6 days average), and at the end of MA treatment (T3, 237.6 days average). Jaw movements were monitored quarterly if amenable to evaluate the stability and effectiveness of treatment.

All patients included in the MA group had conventional pre-therapy orthodontic diagnostic data. Orthodontic intraoral digital images, panoramic radiographs, photographs, study casts, and lateral cephalometric radiography comprised the diagnostic records. CFA (Smartee GS, China) was used for MA therapy in this study. The aligner has an anatomical bite cushion with a built-in reinforcement block on the occlusal surface of the maxillary first molar and the maxillary and mandibular premolar areas, similar to the Twin-Block mechanism, which can achieve the effect of sagittal mandibular protrusion. CFA can also induce horizontal expansion of the dental arch and adjust the tooth position during MA treatment. The patients were instructed to maintain oral hygiene, participate in regular follow-up monitoring, and wear appliances for a total duration of ≥ 20 h per day. The MA treatment phase was discontinued when the following criteria were satisfied: bilateral canine and molar were neutral or slightly mesial, the overbite and overjet of anterior teeth were normal or edge-to-edge, and the mandible could not be retracted to the retrognathic position. The average duration of the MA treatment stage was 237.6 days.

Sample size calculations were performed using G*power (version 3.1; Universität Kiel) with the α = 0.05, β = 0.1, and effect size = 0.8, as described in a previous study[10]. The Mann–Whitney U test was used for the cross-sectional study, and the sample size was calculated as at least 29 subjects in each group. The Wilcoxon sign-rank test was used for the longitudinal study, and the sample size was calculated as at least 16 subjects in each group.

**Fig. 1:**
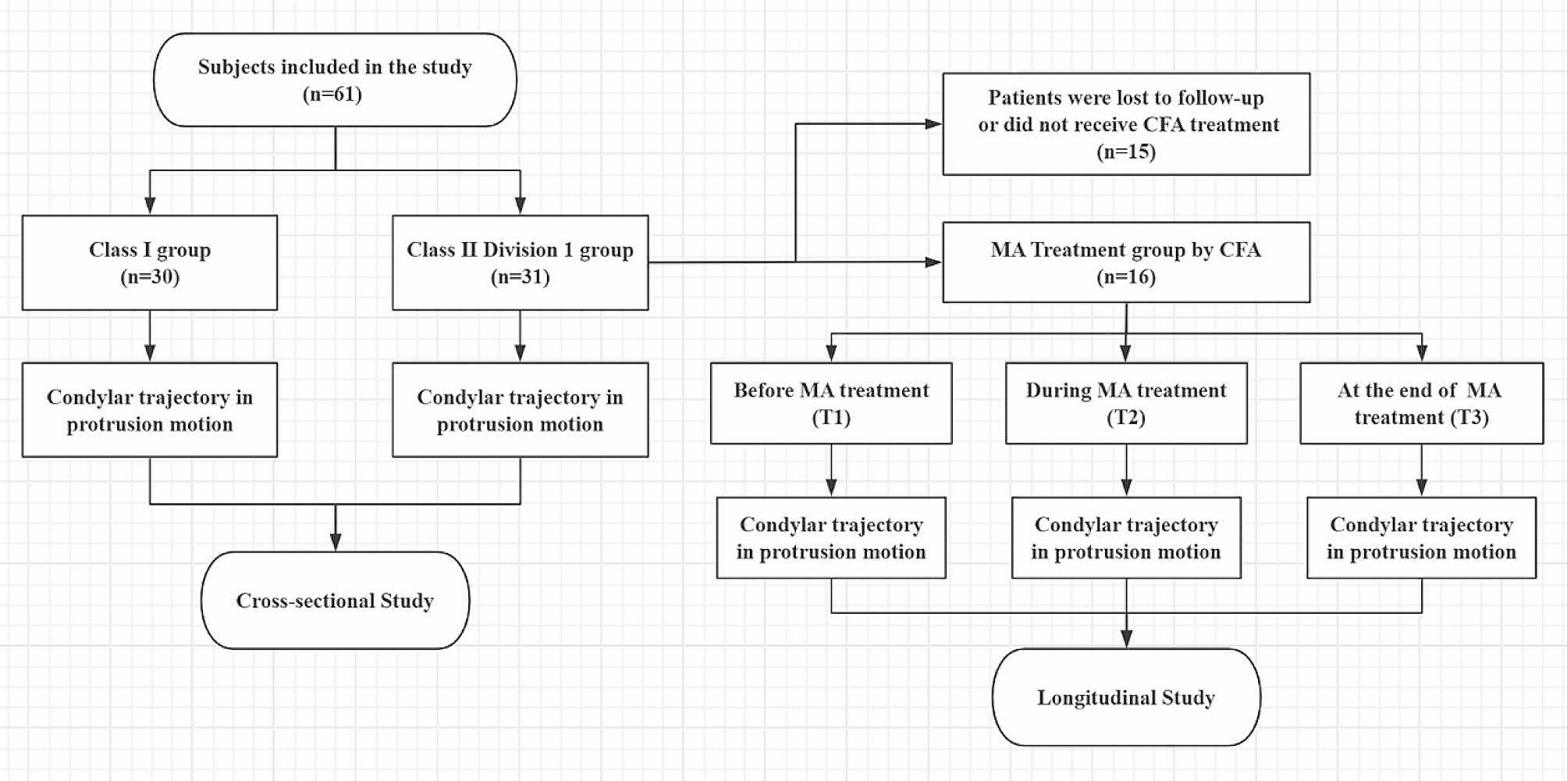
Experimental flow chart. CFA, clear functional aligner; MA, mandibular advancement

The experimental flow chart is presented in Fig. 1.

### Jaw motion analyzer system

**Fig. 2:**
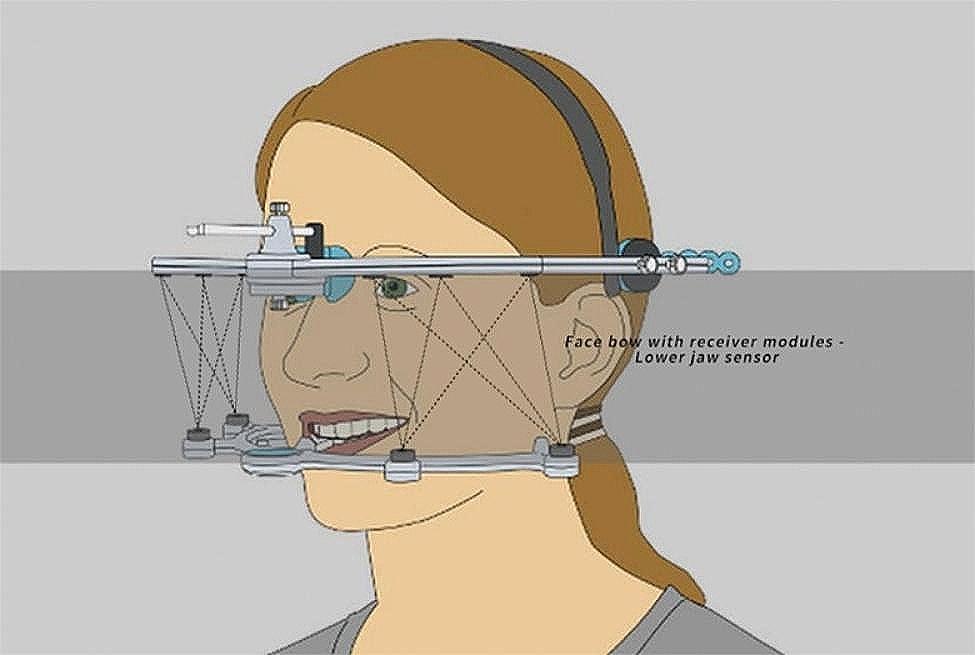
Zebris schematic. Six ultrasonic receivers embedded in the face bow detected sequentially transmitted ultrasound pulses from four ultrasonic emitter arrays embedded on the mandible frame

The mandibular movement was recorded and measured using the JMA^+^ System (Jaw Motion Analyzer^+^, Zebris Medical GmbH, Germany). This motion capture system uses ultrasonic sensing technology to determine the changes in the relative position of the jaw by determining the time required by the ultrasonic pulses to reach the receiver from the transmitter[11]. The device comprises a face bow, metal occlusal bar, mandible frame, and mainframe. The face bow was fixed to the head and face using a headband and nose rest. The metal occlusal bar was bent to match the labial surface of the lower dentition precisely and bonded with a temporization substance (3 M ESPE, Germany) such that the metal bars did not impede the functional motion during typical intercuspation. The mandible frame was fixed to the other end of the metal bar via magnetic suction. The data cable was connected to the mainframe and computer software subsequently, and the device was worn. It emitted continuous ultrasonic pulses through four ultrasound emitter arrays installed on the mandible frame, which were received by six receiver modules integrated into the face bow. The signal was processed by the mainframe, and the jaw movement was recorded, measured, and analyzed using the software (Fig. 2). The precision of this measurement system was rated at 0.1 mm[12].

**Fig. 3:**
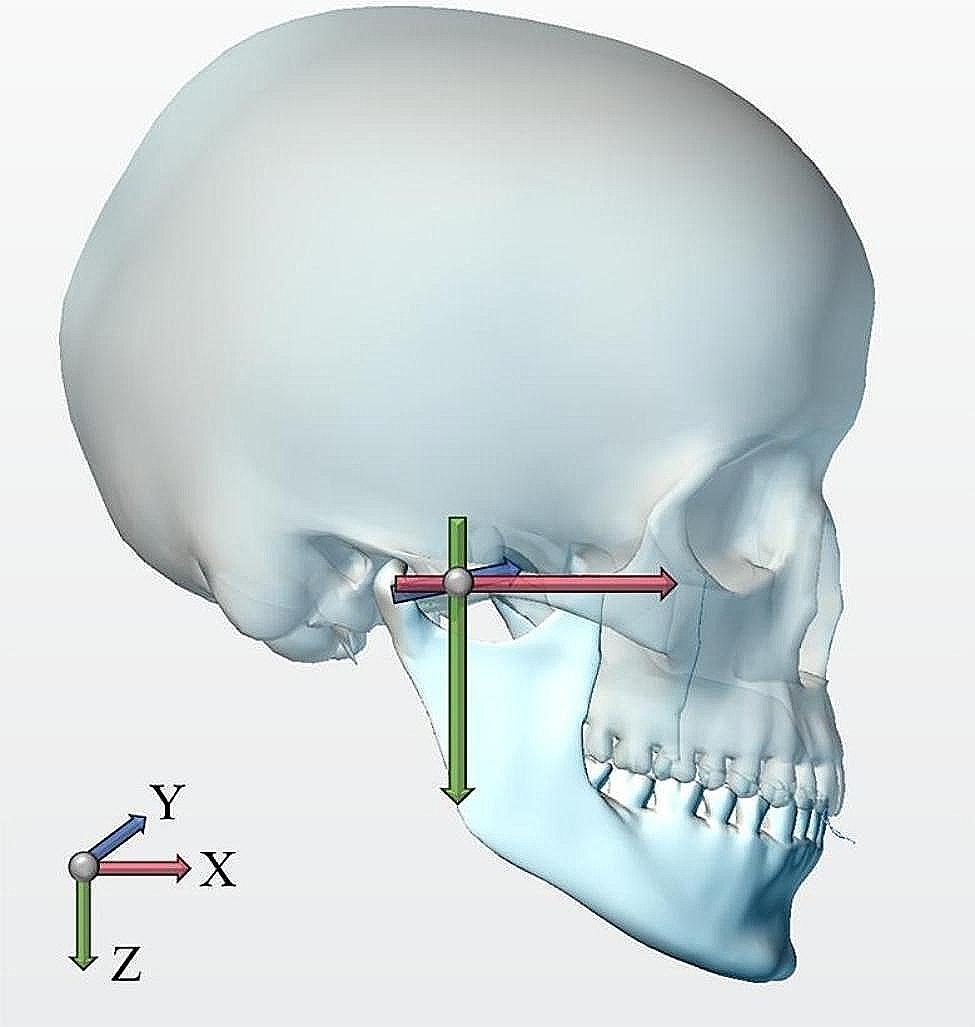
Schematic of the spatial coordinate system. The line connecting the right and left condylar points is the Y axis, with the coordinate origin located in the middle of the condylar. The X-axis represents the anteroposterior direction, whereas the Z-axis represents the superoinferior direction

WinJaw^+^ software (version 1.4.10; Zebris Medical GmbH), which is compatible with the measuring instrument and can establish a digitized three-dimensional spatial coordinate system, was used for data analysis. The midpoint of the line connecting the center points of the condylar movement served as the origin of the three-dimensional spatial coordinate system. In this study, the X-axis was the sagittal anteroposterior direction passing through the origin, the Y-axis was the direction of the line connecting the right and left condylar points, and the Z-axis was the vertical superoinferior directions passing through the origin (Fig. 3).

The participants were instructed to assume the seated position and look forward with their heads unsupported. They were instructed to perform tooth-guided maximum protrusion-retrusion movements at least thrice in this position subsequently. The maximal intercuspation (MI) position was defined as the point at which each movement was commenced and terminated. The examiner did not provide any manual assistance during the motion. All recordings and assessments were performed by the single expert who recorded jaw movements to avoid issues with inter-examiner reliability.

### JMA data processing

The condylar trajectory was used as the mandibular movement assessment index to analyze protrusive movements of the mandible. The anteroposterior displacement (X; in mm) and superoinferior displacement (Z; in mm) of the motion trajectory projected on the sagittal plane, and the left and right displacement (Y; in mm) of the motion trajectory projected on the transverse plane were measured. The maximum displacement in space (S; in mm) was calculated using the following formula:

$$\sqrt{{\text{X}}^{\text{2}}\text{+}{\text{Y}}^{\text{2}}\text{+}{\text{Z}}^{\text{2}}}$$

**Fig. 4:**
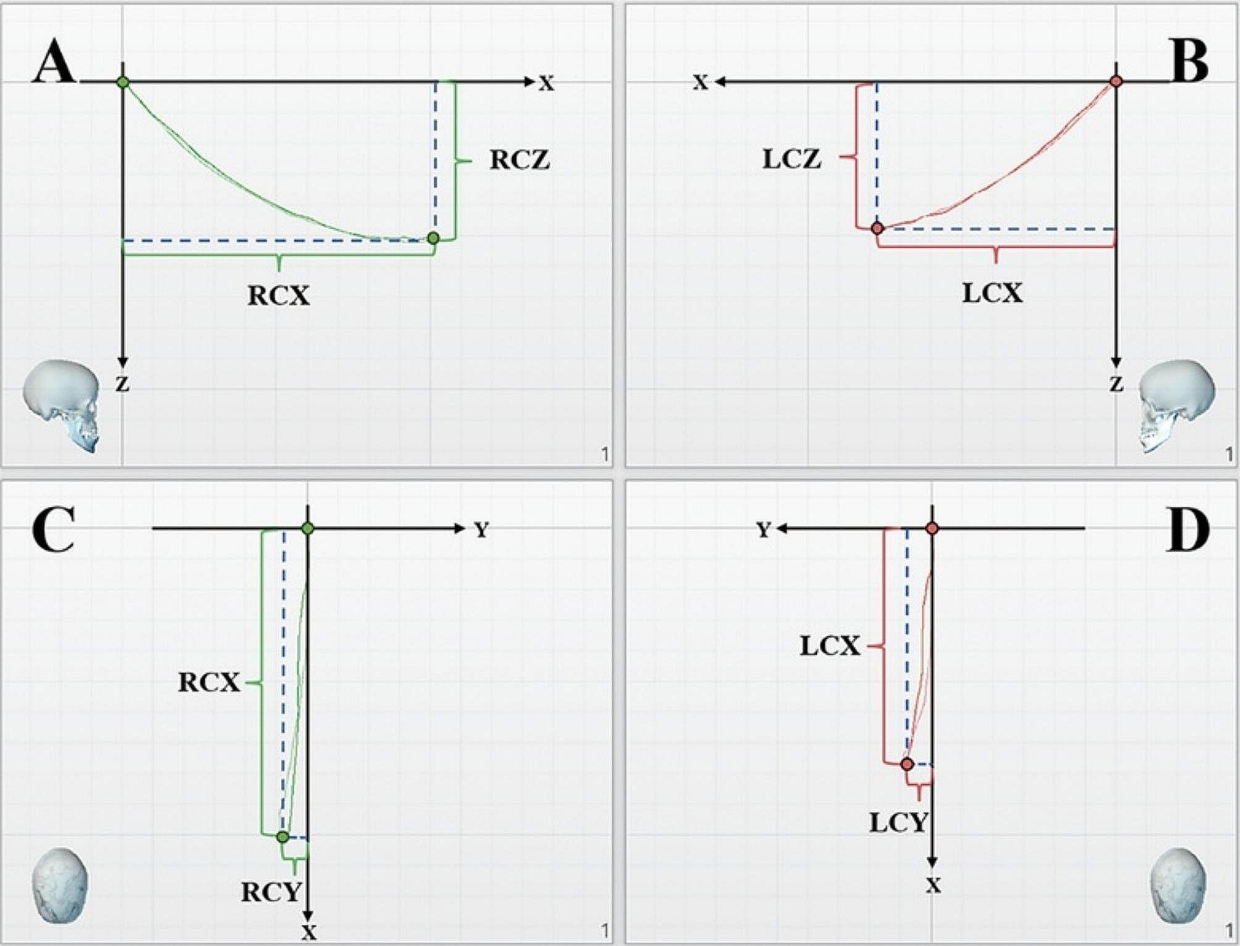
Measurement indices - condyle displacement. **A**: Sagittal projection of the right condyle trajectory; **B**: Sagittal projection of the left condyle trajectory; **C**: Horizontal/transverse projection of the right condyle trajectory; **D**: Horizontal/transverse projection of the left condyle trajectory. RCX/LCX: The right/left condyle maximum anteroposterior displacement on the X-axis. RCY/LCY: The right/left condyle maximum left-right displacement on the Y-axis. RCZ/LCZ: The right/left condyle maximum superoinferior displacement on the Z-axis

**Fig. 5:**
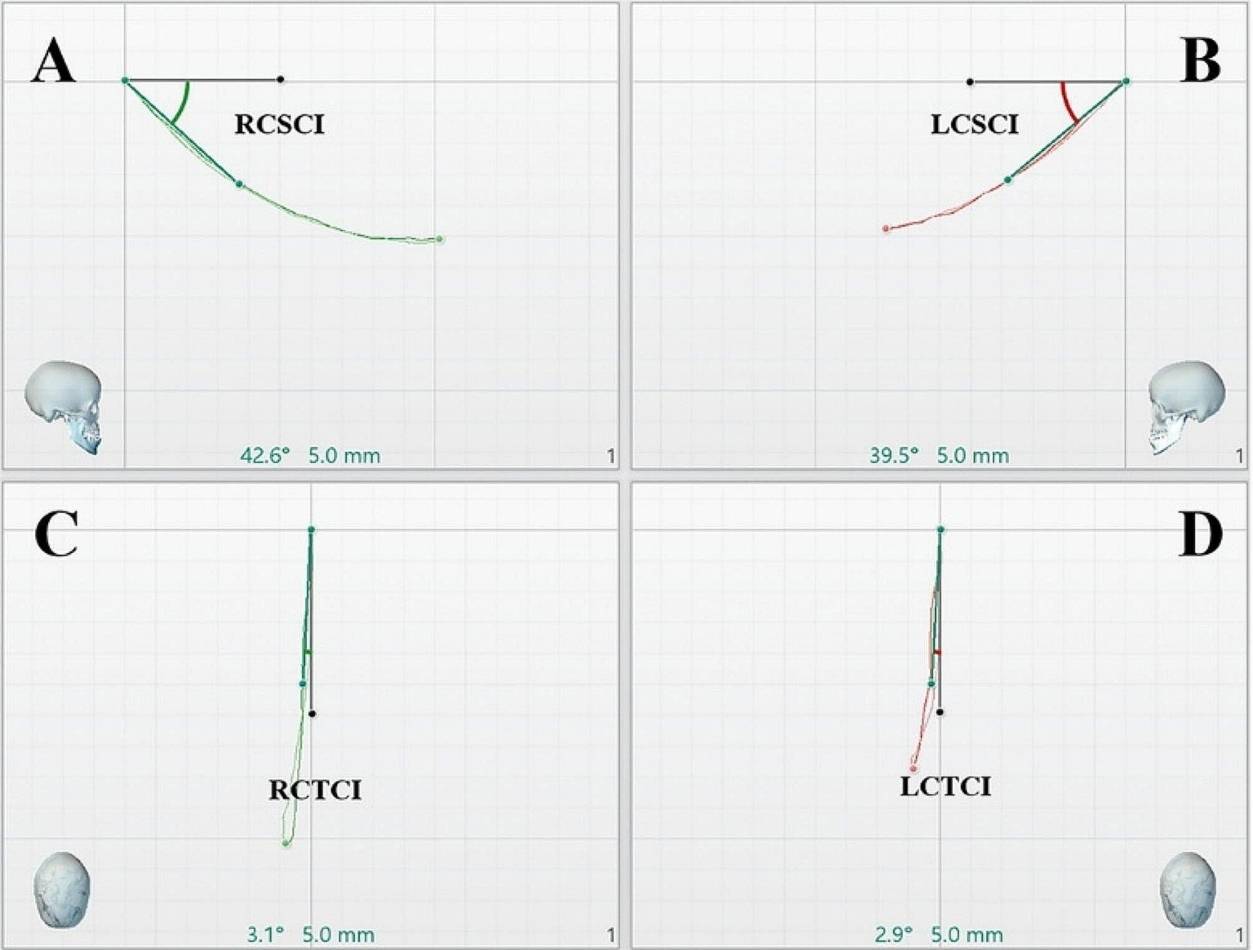
Measurement indices - condylar inclination 5 mm. RCSCI: The right condylar inclination in the sagittal plane when the displacement is 5 mm. LCSCI: The left condylar inclination in the sagittal plane when the displacement is 5 mm. RCTCI: The right condylar inclination in the transverse plane when the displacement is 5 mm. LCTCI: The left condylar inclination in the transverse plane when the displacement is 5 mm

The Y-values for protrusive movement was the absolute value of the left-right excursion of the condyle along the Y-axis. The condyle motion trajectory was projected on the sagittal and the transverse planes, respectively. The angles between the condyle and the X-axis were calculated as the condyle inclination when the displacement was 5mm[13], which were defined SCI and TCI respectively (unit: degrees). Table 1 lists the abbreviations and definitions of jaw movements. Figures 4 and 5 present the measurement methods used.

**Table 1:** Definitions and abbreviations of mandibular movement measures

Abbreviation	Definition
RCX	The right condyle maximum anteroposterior displacement on X-axis
RCY	The right condyle maximum left-right displacement on Y-axis
RCZ	The right condyle maximum superoinferior displacement on Z-axis
RCS	The right condyle maximum space displacement
RCSCI	The right condyle inclination in sagittal plane when displacement 5 mm
RCTCI	The right condyle inclination in transverse plane when displacement 5 mm
LCX	The left condyle maximum anteroposterior displacement on X-axis
LCY	The left condyle maximum left-right displacement on Y-axis
LCZ	The left condyle maximum superoinferior displacement on Z-axis
LCS	The left condyle maximum space displacement
LCSCI	The left condyle inclination in sagittal plane when displacement 5 mm
LCTCI	The left condyle inclination in transverse plane when displacement 5 mm

### Statistical analysis

The age and sex disparities between the groups were assessed using the independent sample T-test and chi-square test, respectively. The results of the normality test of the sample data indicated that the data variables did not entirely follow a normal distribution. Therefore, a non-parametric test was used in the statistical analysis[7]. Variable descriptions are presented as medians and upper and lower quartiles. The data were analyzed using IBM SPSS (version 25.0; IBM Corp., Armonk, NY, USA). The data of the Class II Division 1 and Class I groups were compared using the Mann–Whitney U test in two separate samples. The changes in mandibular movements at T1, T2, and T3 were compared across the treatment stages (T1 vs. T2, T2 vs. T3, and T1 vs. T3) using the Wilcoxon signed-rank test[14]. The pre- and post-treatment groups were compared with the Class I control group in two separate samples (T1 vs. Class I and T3 vs. Class I) using the Mann–Whitney U test. The differences between the right and left lateral condylar movements were assessed using the Wilcoxon signed-rank test. The threshold for statistical significance was chosen at defined as a bilateral α = 0.05 and *P* < 0.05.

To determine the method error, 20 samples were randomly selected, and the correlation of three times protrusive movements or each sample was measured. Intra-class correlation coefficient (ICC) showed good agreement between the measurements (ICC > 0.9).

## Results

### Cross-sectional study

Table 2 presents the sex and age characteristics of the participants. One-way ANOVA (*P* = 0.412) and Pearson’s chi-square test (χ^2^ = 0.957) revealed no discernible differences between the groups in terms of age and sex. Table 3 presents the results of the protrusive movement of the mandible in the Class I and Class II Division 1 groups. The Mann-Whitney U tests revealed that LCX and LCS in the Class II Division 1 group were greater than those in the Class I group [LCX: Class II 8.07(6.77–9.2) mm, Class I 7.57(6-8.64) mm, *P* = 0.004; LCS: Class II 10.12(9.05–11.79) mm, Class I 9.61(8.29–10.85) mm, *P* = 0.009]. Significant differences were observed between the left and right condylar movements in the Class II Division 1 group; however, no significant differences were observed in the Class I group [Class II: LCX 8.07(6.77–9.2) mm, RCX 7.77(6.03–8.5) mm, *P* = 0.031; LCS 10.12(9.05–11.79) mm, RCS 9.66(8.98–10.77) mm, *P* = 0.020]. (Table 4).

**Table 2:** Age and sex distribution in each group

Group	n	Age(years)				Sex	
		Mean	Min	Max	SD	Female	Male
Class I	30	13.8	11	16	1.69	18	12
Class II Division 1	31	13.26	11	16	1.84	18	13
MA Treatment	16	13.25	11	16	1.61	10	6
P Value		0.412				0.957	

Min, minimum; Max, maximum; SD, standard deviation

**Table 3:** Comparison between the results of the condylar movements analysis if the Class I and Class II Division 1 groups

Variables	Class I (*n* = 30)	Class II Division 1 (*n* = 31)	P
RCX (mm)	7.4(6.05–8.48)	7.77(6.03–8.5)	0.228
RCY (mm)	0.62(0.37–0.92)	0.73(0.5–1.07)	0.209
RCZ (mm)	5.6(4.59–7.02)	5.3(4.3–7.3)	0.863
RCS (mm)	9.57(8.5-10.71)	9.66(8.98–10.77)	0.313
RCSCI (°)	47.78(38.03–52.33)	48.4(38-50.97)	0.564
RCTCI (°)	4.62(2.13–7.73)	5.47(2.8–7.63)	0.681
LCX (mm)	7.57(6-8.64)	8.07(6.77–9.2)	0.004**
LCY (mm)	0.63(0.37–0.92)	0.73(0.53–1.03)	0.186
LCZ (mm)	5.92(4.48–7.14)	6.07(4.83–7.27)	0.449
LCS (mm)	9.61(8.29–10.85)	10.12(9.05–11.79)	0.009**
LCSCI (°)	48.32(39.2-53.57)	46.7(35.77–51.23)	0.270
LCTCI (°)	4.27(2.2–6.84)	4.57(2.67–6.27)	0.718

*Note* Values are presented as medians (upper and lower quartiles)

**Statistically significant difference was evaluated by the Mann-Whitney U test, with a significance level of *P* < 0.01

RCX/LCX: The right/left condyle maximum sagittal anteroposterior displacement on the X-axis. RCY/LCY: The right/left condyle maximum left-right displacement on the Y-axis. RCZ/LCZ: The right/left condyle maximum superoinferior displacement on the Z-axis. RCSCI: The right condylar inclination in the sagittal plane when the displacement is 5 mm. LCSCI: The left condylar inclination in the sagittal plane when the displacement is 5 mm. RCTCI: The right condylar inclination in the transverse plane when the displacement is 5 mm. LCTCI: The left condylar inclination in the transverse plane when the displacement is 5 mm

**Table 4:** Comparison between the right and left condylar movements of the Class I and Class II Division 1 groups

Variables	Class I (*n* = 30)			Class II Division 1 (*n* = 31)		
	Left condyle	Right condyle	p	Left condyle	Right condyle	p
LCX - RCX (mm)	7.57(6-8.64)	7.4(6.05–8.48)	0.546	8.07(6.77–9.2)	7.77(6.03–8.5)	0.031*
LCY - RCY (mm)	0.63(0.37–0.92)	0.62(0.37–0.92)	0.816	0.73(0.53–1.03)	0.73(0.5–1.07)	0.878
LCZ - RCZ (mm)	5.92(4.48–7.14)	5.6(4.59–7.02)	0.695	6.07(4.83–7.27)	5.3(4.3–7.3)	0.096
LCS - RCS (mm)	9.61(8.29–10.85)	9.57(8.5-10.71)	0.373	10.12(9.05–11.79)	9.66(8.98–10.77)	0.020*
LCSCI – RCSCI (°)	48.32(39.2-53.57)	47.78(38.03–52.33)	0.643	46.7(35.77–51.23)	48.4(38-50.97)	0.652
LCTCI – RCTCI (°)	4.27(2.2–6.84)	4.62(2.13–7.73)	0.524	4.57(2.67–6.27)	5.47(2.8–7.63)	0.118

*Note* Values are presented as medians (upper and lower quartiles)

*Statistically significant difference was evaluated by the Wilcoxon signed-rank test, with a significance level of *P* < 0.05

RCX/LCX: The right/left condyle maximum sagittal anteroposterior displacement on the X-axis. RCY/LCY: The right/left condyle maximum left-right displacement on the Y-axis. RCZ/LCZ: The right/left condyle maximum superoinferior displacement on the Z-axis. RCSCI: The right condylar inclination in the sagittal plane when the displacement is 5 mm. LCSCI: The left condylar inclination in the sagittal plane when the displacement is 5 mm. RCTCI: The right condylar inclination in the transverse plane when the displacement is 5 mm. LCTCI: The left condylar inclination in the transverse plane when the displacement is 5 mm

### Longitudinal study

**Fig. 6:**
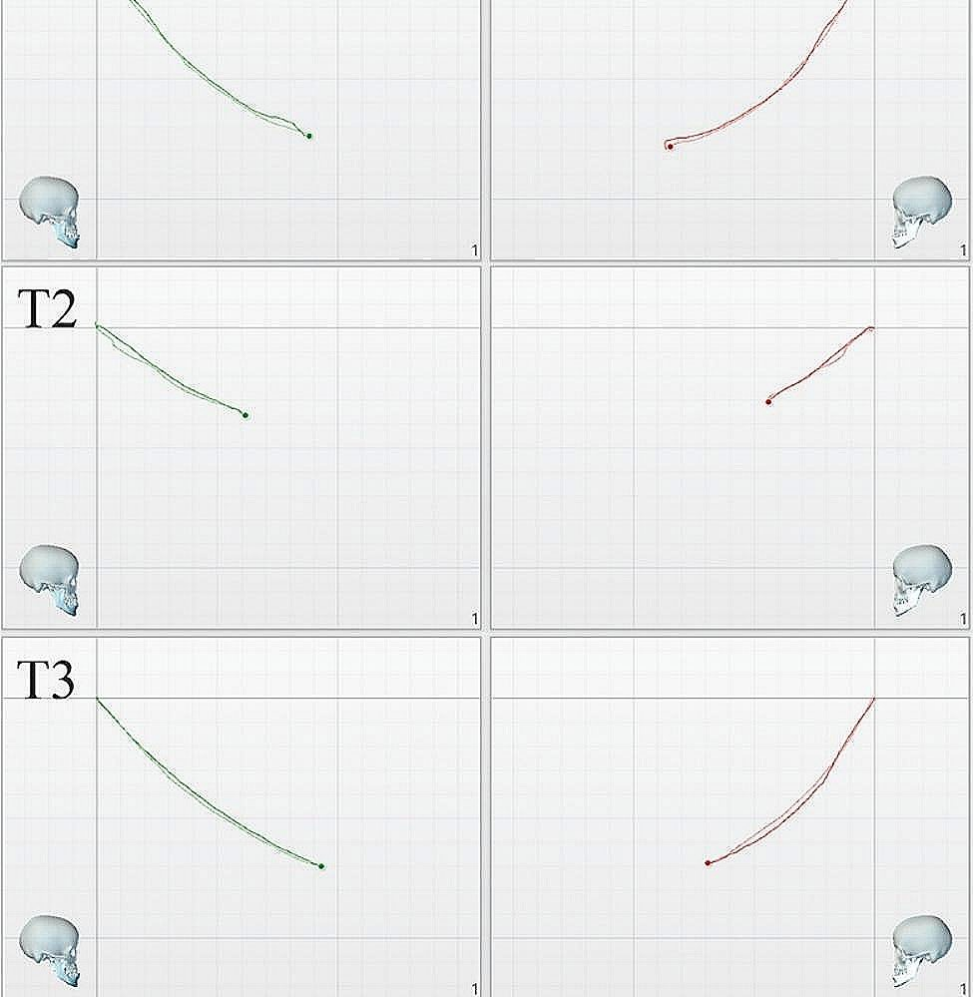
Projections of the condylar trajectories in the sagittal plane during protrusive movement of the mandible in a patient at different stages of MA treatment using CFA. **T1**: Pre-treatment; **T2**: 82 days during MA; **T3**: 222 days (end of MA). CFA, clear functional aligner; MA, mandibular advancement

**Fig. 7:**
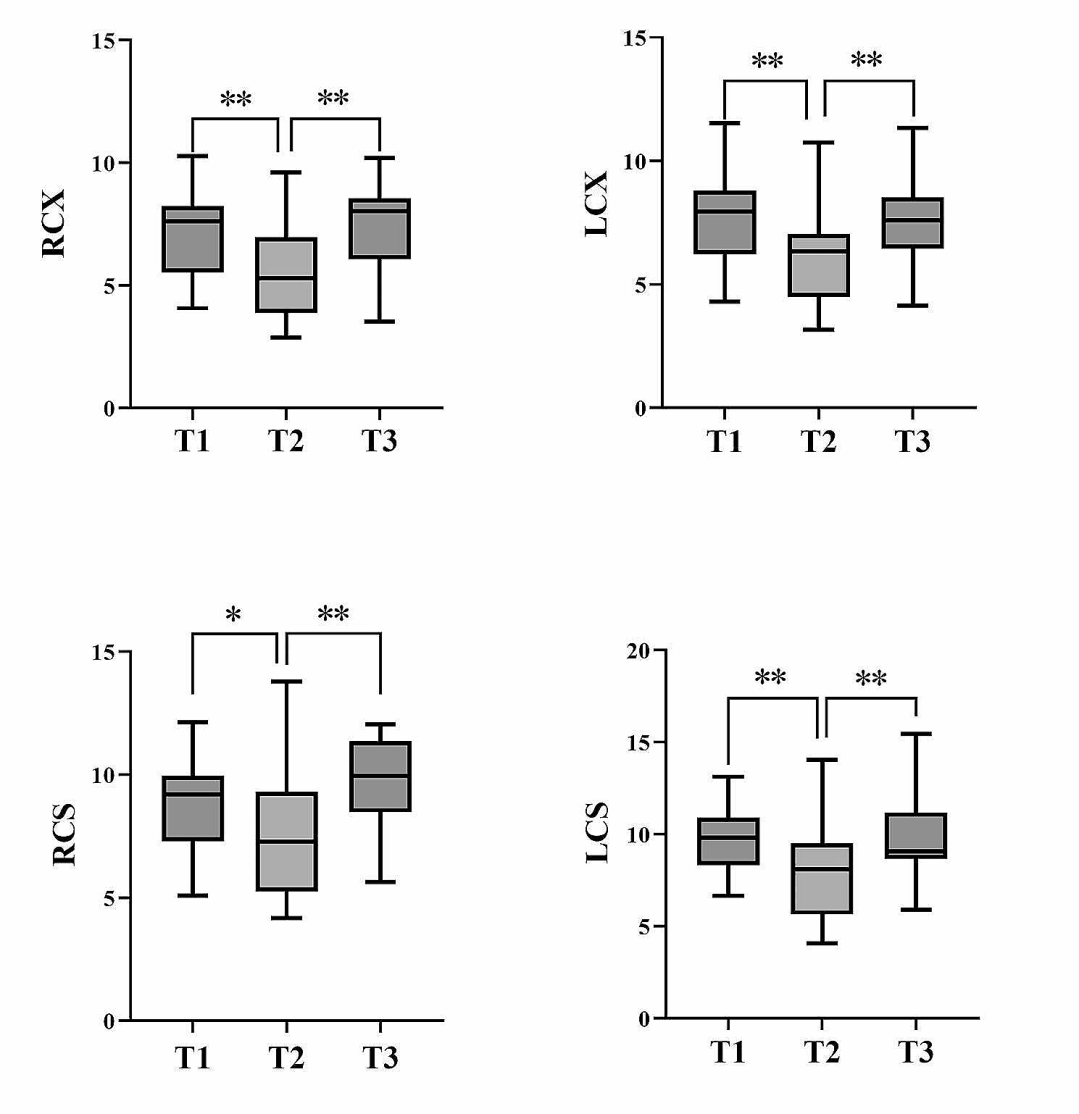
Box line plots of the changes in the anteroposterior displacement and space displacement at different treatment stages during protrusion. **T1**: Pre-treatment, **T1**: during MA treatment (105.6 days average), **T1**: at the end of MA treatment (237.6 days average). RCX/LCX: The right/left condyle maximum anteroposterior displacement on the X-axis. RCX/LCX: The right/left condyle maximum space displacement on the X-axis. *Statistically significant at *P* < 0.05, **Statistically significant at *P* < 0.01

Table 5 provides an overview of the longitudinal data of the regarding the changes in condylar mobility during MA treatment in the Treatment group. The anteroposterior displacement, as well as the space displacement of the condyle, during the protrusive movement decreased at T2 and then increased to the same level as that at T1 at T3. The difference was statistically significant. The values for T3 and T1 were comparable. Figure 6 depicts the projections of the condylar trajectories in the sagittal plane during protrusive movement of the mandible in a patient at different stages of MA treatment using CFA. Figure 7 depicts the box-line plots of the changes in the anteroposterior displacement and space displacement at different treatment stages during protrusion. Wilcoxon signed-rank tests of the left and right condyles exhibited significant differences in LCX-RCX and LCS-RCS at T1; however, the left and right condyles did not vary significantly from one another at T2 and T3 (Table 6).

**Table 5:** Longitudinal changes in the condylar movements during MA using CFA in the treatment groups

Variables	T1 (*n* = 16)	P-value (T1VST2)	T2 (*n* = 16)	P-value (T2VST3)	T3 (*n* = 16)	P-value (T1VST3)
RCX (mm)	7.62(5.52–8.24)	0.004**	5.28(3.86–6.97)	0.004**	8.03(6.07–8.56)	0.756
RCY (mm)	0.78(0.54–1.18)	0.379	0.72(0.6–0.83)	0.955	0.75(0.47–1.21)	0.691
RCZ (mm)	5.05(4.11–6.25)	0.796	5(3.34–6.54)	0.036*	5.82(4.43–6.74)	0.049*
RCS (mm)	9.2(7.28–9.95)	0.039*	7.27(5.24–9.3)	0.004**	9.95(8.46–11.36)	0.196
RCSCI (°)	42.52(35.19–49.19)	0.365	45.4(35.88–51.43)	0.134	44.7(41.2-52.34)	0.079
RCTCI (°)	5.67(3.29–6.78)	0.326	7(4.55–8.25)	0.501	6.33(2.48–12.87)	1.000
LCX (mm)	7.95(6.22–8.79)	0.007**	6.32(4.48–7.05)	0.006**	7.58(6.43–8.53)	0.196
LCY (mm)	0.78(0.58–1.13)	0.408	0.68(0.6–0.9)	0.587	0.73(0.51–0.95)	0.394
LCZ (mm)	5.25(3.79–6.55)	0.737	4.75(3.49–6.65)	0.121	5.88(4.55–6.63)	0.278
LCS (mm)	9.8(8.31–10.9)	0.013*	8.1(5.64–9.49)	0.023*	9.07(8.65–11.19)	0.326
LCSCI (°)	41.1(32.84–49.07)	0.469	38.23(32.83–49.89)	0.408	43.17(39.15–49.78)	0.836
LCTCI (°)	4.85(2.77–6.77)	0.301	5.65(4.61–10.94)	0.535	4.9(3.2–9.18)	0.796

*Note* Values are presented as medians (upper and lower quartiles)

**Statistically significant difference was evaluated by the Wilcoxon signed-rank test, with a significance level of *P* < 0.01

*Statistically significant difference was evaluated by the Wilcoxon signed-rank test, with a significance level of *P* < 0.05

CFA, clear functional aligner; MA, mandibular advancement. RCX/LCX: The right/left condyle maximum sagittal anteroposterior displacement on the X-axis. RCY/LCY: The right/left condyle maximum left-right displacement on the Y-axis. RCZ/LCZ: The right/left condyle maximum superoinferior displacement on the Z-axis. RCSCI: The right condylar inclination in the sagittal plane when the displacement is 5 mm. LCSCI: The left condylar inclination in the sagittal plane when the displacement is 5 mm. RCTCI: The right condylar inclination in the transverse plane when the displacement is 5 mm. LCTCI: The left condylar inclination in the transverse plane when the displacement is 5 mm

**Table 6:** Comparison between the right and left condylar movements of the treatment groups

Variables	T1 (*n* = 16)			T2 (*n* = 16)			T3 (*n* = 16)		
	Left condyle	Right condyle	P	Left condyle	Right condyle	P	Left condyle	Right condyle	P
LCX – RCX (mm)	7.95(6.22–8.79)	7.62(5.52–8.24)	0.049*	6.32(4.48–7.05)	5.28(3.86–6.97)	0.196	7.58(6.43–8.53)	8.03(6.07–8.56)	0.796
LCY – RCY (mm)	0.78(0.58–1.13)	0.78(0.54–1.18)	0.574	0.68(0.6–0.9)	0.72(0.6–0.83)	0.859	0.73(0.51–0.95)	0.75(0.47–1.21)	0.778
LCZ – RCZ (mm)	5.25(3.79–6.55)	5.05(4.11–6.25)	0.088	4.75(3.49–6.65)	5(3.34–6.54)	0.979	5.88(4.55–6.63)	5.82(4.43–6.74)	0.535
LCS – RCS (mm)	9.8(8.31–10.9)	9.2(7.28–9.95)	0.020*	8.1(5.64–9.49)	7.27(5.24–9.3)	0.148	9.07(8.65–11.19)	9.95(8.46–11.36)	0.918
LCSCI – RCSCI (°)	41.1(32.84–49.07)	42.52(35.19–49.19)	0.877	38.23(32.83–49.89)	45.4(35.88–51.43)	0.115	43.17(39.15–49.78)	44.7(41.2-52.34)	0.196
LCTCI – RCTCI (°)	4.85(2.77–6.77)	5.67(3.29–6.78)	0.485	5.65(4.61–10.94)	7(4.55–8.25)	0.836	4.9(3.2–9.18)	6.33(2.48–12.87)	0.326

*Note* Values are presented as medians (upper and lower quartiles);

*Statistically significant difference was evaluated by the Wilcoxon signed-rank test, with a significance level of *P* < 0.05

RCX/LCX: The right/left condyle maximum sagittal anteroposterior displacement on the X-axis. RCY/LCY: The right/left condyle maximum left-right displacement on the Y-axis. RCZ/LCZ: The right/left condyle maximum superoinferior displacement on the Z-axis. RCSCI: The right condylar inclination in the sagittal plane when the displacement is 5 mm. LCSCI: The left condylar inclination in the sagittal plane when the displacement is 5 mm. RCTCI: The right condylar inclination in the transverse plane when the displacement is 5 mm. LCTCI: The left condylar inclination in the transverse plane when the displacement is 5 mm

Consistent with the findings of cross-sectional study, the MA treatment at T1 exhibited greater LCX during protrusive movement than those of the Class I group. At the end of MA treatment(T3), the amount of anteroposterior condylar displacement and the space displacement were close to those of Class I but still slightly larger (Table 7).

**Table 7:** Comparison of condylar motion analysis between the Class I and MA treatment group at T1 and T2

Variables	T1 (*n* = 16)	P (T1VS Class I)	Class I (*n* = 30)	P (Class I VST3)	T3 (*n* = 16)
RCX (mm)	7.62(5.52–8.24)	0.686	7.4(6.05–8.48)	0.344	8.03(6.07–8.56)
RCY (mm)	0.78(0.54–1.18)	0.079	0.62(0.37–0.92)	0.103	0.75(0.47–1.21)
RCZ (mm)	5.05(4.11–6.25)	0.109	5.6(4.59–7.02)	0.818	5.82(4.43–6.74)
RCS (mm)	9.2(7.28–9.95)	0.747	9.57(8.5-10.71)	0.321	9.95(8.46–11.36)
RCSCI (°)	42.52(35.19–49.19)	0.062	47.78(38.03–52.33)	0.712	44.7(41.2-52.34)
RCTCI (°)	5.67(3.29–6.78)	0.636	4.62(2.13–7.73)	0.289	6.33(2.48–12.87)
LCX (mm)	7.95(6.22–8.79)	0.029*	7.57(6-8.64)	0.097	7.58(6.43–8.53)
LCY (mm)	0.78(0.58–1.13)	0.072	0.63(0.37–0.92)	0.221	0.73(0.51–0.95)
LCZ (mm)	5.25(3.79–6.55)	0.482	5.92(4.48–7.14)	0.800	5.88(4.55–6.63)
LCS (mm)	9.8(8.31–10.9)	0.174	9.61(8.29–10.85)	0.249	9.07(8.65–11.19)
LCSCI (°)	41.1(32.84–49.07)	0.117	48.32(39.2-53.57)	0.185	43.17(39.15–49.78)
LCTCI (°)	4.85(2.77–6.77)	0.564	4.27(2.2–6.84)	0.235	4.9(3.2–9.18)

*Note* Values are presented as medians (upper and lower quartiles)

*Statistically significant difference was evaluated by the Mann-Whitney U test, with a significance level of *P* < 0.05

RCX/LCX: The right/left condyle maximum sagittal anteroposterior displacement on the X-axis. RCY/LCY: The right/left condyle maximum left-right displacement on the Y-axis. RCZ/LCZ: The right/left condyle maximum superoinferior displacement on the Z-axis. RCSCI: The right condylar inclination in the sagittal plane when the displacement is 5 mm. LCSCI: The left condylar inclination in the sagittal plane when the displacement is 5 mm. RCTCI: The right condylar inclination in the transverse plane when the displacement is 5 mm. LCTCI: The left condylar inclination in the transverse plane when the displacement is 5 mm

## Discussion

Jaw motion analysis has been used increasingly in both clinical and research applications, such as measurement of dynamic occlusion assisted in fabrication prosthesis [15] and assessing the progression and outcome of treatments[16]. Moreover, it has also been employed intra-operatively to guide TMJ surgery[17]. Jaw kinematics measurement has played an important role in providing an objective basis for diagnosing musculoskeletal disorders of the jaw[18]. Motion measurement techniques depend on the ease of use, minimal invasiveness, and rapid data collection capability in clinical settings. Commercial ultrasonic tracking equipment has gained popularity as it is faster and simpler to deploy, requires minimal calibration, and employs lightweight face-bows that do not impede natural mandibular motion. Therefore, the JMA^+^ system based on the ultrasonic principle was selected as the research instrument.

The JMA^+^ system, combined with the WinJaw^+^ software, can track and record the three-dimensional movements of the incisal reference point and the two condylar kinematic centers. WinJaw^+^ version 1.4.10 can automatically calculate the linear distance motion of the incisor during opening and laterotrusion (frontal projection) and protrusion (transverse projection), as well as the actual condylar path length in the sagittal plane during opening movement[8, 19]. TMJ is a bilateral synovial joint between the skull and the mandible. The condyle and the articular disc form the condyle-articular disc complex comprising the attached ligaments, muscles, and joint capsule. The motion of the condyle is essential for normal mandibular function and maintaining the quality of life[20]. Moreover, the condylar trajectory reflects the functional coordination of this complex under the control of the central nervous system. Most previous studies directly used the values automatically calculated by software to analyze the motion parameters.^8,19,21^ However, the condylar trajectory was placed in the three-dimensional space coordinate system, and the condylar displacement and inclination were analyzed from the anteroposterior, horizontal, and vertical directions using the measuring ruler of the software in the present study to conduct a more in-depth study of the motion trajectory.

### Cross-sectional study

Significant kinematic variations were observed between individuals with untreated Class I and Class II Division 1 malocclusion in the cross-sectional study, particularly in terms of LCX and LCS, where statistically significant differences were evident. The Class II Division 1 group displayed notable disparities in terms of the left and right condyle measurements, as revealed by the Wilcoxon signed-rank test.

### Anatomical evidence

The changes in the position of the condyle in the articular fossa, articular space, and articular disk have been prominent research subjects in the domain of TMJ. However, the results of studies on the placement of Class II Division 1 condyles have been inconsistent. Vitral et al[22]. conducted an evaluation of the concentric position of the condyle in patients with Class II Division 1 subdivision malocclusions and demonstrated that the relative articular fossa exhibited non-concentric positioning for sides I and II and that side II exhibited a statistically significant anteriorly positioned condyle. Jacob et al[23]. discovered that compared with adolescents with Class I malocclusion, those with Class II malocclusion exhibit lower vertical condyle development and glenoid fossa modeling. Thus, individuals with Class II malocclusion may exhibit lower mandibular development compared with those with Class I malocclusion. Although differences were observed between Class I and II malocclusions in terms of the condylar position, studies by Arieta-Miranda et al[24]. and Cohlmia et al[25]. revealed that these differences may not be clinically significant, given the remaining normal physiological position of the condyle in the articular fossa.

Based on the abovementioned static anatomical evidence, it was speculated that the difference between the Class I and Class II groups may be attributed to the anatomical differences in the joint structure in the present study. However, such anatomical differences are minor. Previous studies have reported inconsistent data, and it is unclear whether these differences will eventually cause changes in functional kinematics.

### Functional evidence

Green[26] asserted that the condyle moves in a back-and-forth manner along the articular eminence, akin to a small ball rolling over the crest of a hill. According to this perspective, the maxillomandibular position relationship, as well as the equilibrium of the masticatory system, typically falls within the physiologically acceptable range in individuals with healthy dentition. This may indicate that no significant differences are present between individuals with Class I and Class II malocclusion without joint problems in terms of mandibular movement.

In contrast, Zimmer et al[7]. reported that the Class II group exhibited a substantial tendency toward a higher mandibular movement capacity than the Class I group for protrusive motion. Similar disparities in protrusion were also observed by Tuncer et al[27]. among in adolescents. Satygo et al[28]. reported that Class II malocclusions involves temporalis and masseter muscle activity during clenching, which is almost 1.5 times lower than that observed in individuals with Class I malocclusions.

The abovementioned functional evidence suggests that the trend for greater anteroposterior displacement and the space displacement in Class II participants observed in the present study is consistent with the findings of the studies conducted by Zimmer et al[7]. and Tuncer et al[27]. Zimmer et al[7]. reported that the ANB angle and overjet are correlated with mandibular protrusion mobility; however, this functional difference cannot be explained by a single factor alone, and is more likely to be the result of a complex combination of multiple factors, such as the TMJ, neuromuscular function, ligaments, and other factors that can alter the capacity of the TMJ to protrude. Therefore, we hypothesized that adolescents with Class II Division 1 malocclusion require more compensation during the protrusion of functional movements due to the presence of a retrognathic mandible. This is demonstrated by the increased anteroposterior and space displacement movement during protrusion movements, which reflects adaptive changes in neuromuscular function in the sagittal direction with increased mobility.

The condylar inclination was evaluated when the condyle motion was 5 mm in the present study, as described by Slavicek[13]. Minimal differences were observed between the SCI and TCI values of individuals with Class I and Class II, consistent with the findings of previous studies[29, 30].

Statistically significant differences were also observed in the Class II group in terms of the LCX-RCX and LCS-RCS values during protrusion. However, no significant differences were observed between the left and right condylar movement tracings of the Class I group. The Wilcoxon signed-rank test performed to evaluate the left and right condylar movement trajectory within the group reflected the differences between the bilateral condyles during symmetrical mandibular movement. This indicates that the left and right condyles of individuals with Class II malocclusion demonstrated higher asymmetry than those of individuals with Class I malocclusion during protrusive motion. However, Cohlmia et al[25]. hypothesized that the asymmetrical location of the condyles was a characteristic feature in the general populace. Normal condylar placement in normal joints can exhibit significant variance. And Blaschke et al. [31] reported that these spatial variations might not be clinically meaningful. In contrast, the study by Antonarakis et al. [32] on facial expressions revealed that adolescents with Class II Division 1 malocclusion exhibited greater asymmetry in mouth width during repose and while smiling than adolescents with Class I malocclusion. The facial expression and activity of the masticatory muscles in the cephalofacial region are related to neuromuscular control functions. Therefore, it was hypothesized that the asymmetry of condylar movement observed in individuals with Class II compared with that in individuals with Class 1 malocclusion may be caused by neuromuscular dysfunction.

In summary, adolescents with Class II Division 1 malocclusion had higher protrusive capacity than those with Class I, which reflects adaptive changes in neuromuscular function in the sagittal direction with increased mobility.

### Longitudinal study

The condylar anteroposterior displacement and space displacement during protrusive movement decreased at T2, and then increased at T3 during MA treatment in the longitudinal study. The value at T3 was comparable with or slightly greater than that at T1. A significant difference between the left and right condyle movement was observed at the T1 stage; however, no significant difference was observed between the left and right condyle movement at the T2 and T3 stages.

### Anatomical evidence

In terms of osseous remodeling and condylar location during MA, Ruf and Pancherz[33, 34] investigated the use of magnetic resonance imaging techniques for assessing condyle and TMJ fossa remodeling with magnetic resonance images obtained prior to therapy, at the start of MA, 6–12 weeks following MA, and at the completion of treatment. A modification of the posterosuperior region of the condyle was observed during MA treatment at 6–12 weeks in young adults and teenagers. Kinzinger et al[35]. reported that the condyles are displaced significantly anteriorly during early MA treatment and eventually returned to a central location within the fossa after the appliance was removed. These researchers believed that MA treatment resulted in remodeling of the condyles and fossae, with changes in their relative positions.

Pancherz et al[36, 37]. observed a protruding disc position prior to MA therapy and a more retrusive position during MA treatment. The discs were restored to their original location or retracted relative to their initial location after MA. Aidar et al[38]. reported that the articular disc position was normal in all TMJs prior to commencing treatment, moved posteriorly during MA therapy, and was restored to the normal position following treatment. These researchers concluded that MA therapy restored the articular disc to its normal functional position even when it was originally displaced anteriorly.

It was speculated that the differences observed in the present study could be attributed to the changes in the condylar and articular disc positions during early treatment based on the abovementioned anatomical evidence. The condyle and articular disc returned to their normal functional or original positions as the duration of treatment increased. Therefore, the condylar anteroposterior displacement and space displacement in protrusive movement decreased at T2 compared with that at T1 and returned to the pre-treatment levels or were even larger at T3. This trend was more evident for anterior and posterior displacement, as MA treatment is more closely related to the changes in the sagittal position of the mandible.

### Functional evidence

Specific neuromuscular and skeletal changes caused by experimental conditions were observed during 13 weeks of MA treatment in an animal study. For instance, the activity of the lateral pterygoid gradually increased during functional movements and then during the maintenance of mandibular postural position. However, this activity decreased by the end of the experiment[39] and most experimental animals exhibited an anteroposterior alteration in molar relationship at the end of the experimental period. This temporal relationship between the onset and cessation of abnormal neuromuscular function and the restoration of skeletal equilibrium suggests that lesser neuromuscular compensation is required as the mandible undergoes adaptive remodeling[39]. Aggarwal et al[40]. reported a significant increase in the activity of the masseter muscle from the beginning of treatment to 3 and 6 months into the therapy. The most significant change in EMG activity was noted at the 3-month mark, which coincided with substantial improvements in sagittal mandibular and maxillary relationships in the patients. Sood et al[41]. revealed that neuromuscular adaptations following 6 months of FFA treatment remained consistent over a monitoring period of 24 months, suggesting that maxillofacial neuromuscular function tends to stabilize after 6 months of MA treatment.

Greene[26] suggested that the neuromuscular program “ENGRAM”, which is defined as “a presumed encoding in neural tissue that provides a physical basis for the persistence of memory,”[42] governs regular MI associations. The ability of the mandible to open, close, chew food, and perform other activities is governed by the engram associated with the human jaw. The MI occlusal relationship was blocked by the functional appliance, and the mandible was guided to the target position to establish a new jaw relationship, thereby deprogramming the initial neuromuscular program. Okeson[43] defined a healthy masticatory system as a stable occlusal position working in unison with a steady joint position. To optimize masticatory function over the course of the lifetime of the patient, it is crucial to establish a lifelong optimal masticatory system to achieve a stable and harmonious relationship between the dental and skeletal positions and reduce the risk factors for temporomandibular disorders in patients. Thus, although functional orthodontic treatment disrupts the initial neuromuscular equilibrium, it is important to work within the physiological tolerance of the patient, and achieve a neuromuscular equilibrium that is established once the jaw has been stabilized and the bone has undergone remodeling. Future functional issues can be prevented from arising by facilitating functional orthodontic therapy among developing youngsters to assist them in establishing occlusion in the position of musculoskeletal stability. Therefore, rational functional orthodontic treatment with appropriate indications is beneficial to the growth and development of the patient, as well as the functional health of the stomatognathic system.

The present study aimed to identify the reasons contributing to this discrepancy based on the abovementioned functional evidence. The treatment altered the tooth position and intermaxillary relationships at T2, resulting in unstable occlusion. Modifications in the TMJ region were most active during this stage, necessitating more neuromuscular function to compensate for the initial deprogramming of neuromuscular balance, which manifested as a reduction in the sagittal range of motion during mandibular movement. However, this decrease was temporary. A more stable clinical sagittal maxillo-mandibular relationship of anterior mandibular displacement was observed as the treatment progressed to the end of MA (T3), and most patients were unable to regress to the pre-treatment position. The strength of the alterations in the TMJ region diminished, compensation for neuromuscular function decreased, and the neuromuscular equilibrium was restored, as evidenced by the significant increase in the sagittal range of motion of the condyles. Thus, it was concluded that the changes in mandibular sagittal mobility were generally consistent with the improvements in clinical maxillomandibular sagittal relationships and that neuromuscular function remained stable during MA treatment.

The experimental findings also revealed that the condylar anteroposterior displacement and space displacement at T3 were still slightly larger. Consistent with the findings of previous studies[6, 8], neuromuscular memory function was found to be difficult to change; thus, patients may unconsciously return to their original movement habits. It could also be that the neuromuscular system takes longer to adapt compared with dental adjustment.

The present study demonstrated a statistically significant difference between the left and right condylar movements before CFA therapy; however, this difference disappeared at T2 and T3. Therefore, it was speculated that the condylar movement symmetry increased after CFA treatment. These findings are consistent with the results obtained by Antonarakis and Kiliaridis[32], who revealed a trend toward greater symmetry after MA treatment compared with pre-treatment conditions. Previous occlusal studies revealed that the occlusal interference was reduced and that the occlusion became more balanced and stable in patients with class II malocclusion after orthodontic treatment[44]. These findings may be attributed to the muscle position becoming harmonious with the tooth position after MA treatment, which is conducive to more symmetrical left and right condylar movement. Moreover, CFA can also initially align the teeth and adjust any midline deviations, in addition to correcting sagittal mismatch.

The present study investigated the mandibular motion, neuromuscular function, and their relationship in the context of Class II Division 1 malocclusion and orthodontic treatment with CFA. The findings of this study highlight the importance of monitoring neuromuscular adaptation during early orthodontic intervention, which could contribute to our understanding of the effect of orthodontic interventions on the functional occlusal systems, and suggests that proper treatment can help establish a stable and balanced occlusal system. Nevertheless, this study has some limitations. First, the observation period of existing study is only a phase and should encompass the full cycle of functional orthodontics and orthodontic treatment, and electromyography monitoring would be more comprehensive if it is combined with the corresponding process. Second, the sample size of the present study was limited and further large-scale studies must be conducted to provide more conclusive findings. Follow-up observations are currently being recorded by our treatment team to strengthen and validate these findings.

## Conclusions

1. Adolescents with mandibular retrognathia of Class II Division 1 malocclusion possess increased capacity for protrusive movement. The left and right condylar movement of Class II Division 1 malocclusion differ significantly from those of Class I.
2. The condylar range of motion decreased first and then increased during MA treatment with CFA, which may be an adaptive response of neuromuscular function to jaw position changes.
3. The difference between the left and right condylar movement decreased and the symmetry increased after MA treatment with CFA.
4. This study emphasizes the significance of evaluating the neuromuscular adaptation and TMJ status of the patient during the early stages of functional orthodontic treatment (approximately 3 months) for the early detection and prevention of possible risks of TMJ disorders.

## Data Availability

The datasets used and analyzed during the current study available from the corresponding author on reasonable request.

## Abbreviations

MA: Mandibular advancement
FFA: Fixed-function appliance
RFA: Removable-function appliance
CAD: Computer-aided design
CAM: Computer-aided manufacturing
3D: Three-dimensional
CFA: Clear functional aligner
TMJ: Temporomandibular joint
JMA: Jaw motion analyzer
MI: Maximal intercuspation
EMG: Electromyography
